# 2-[(*E*)-2-(Nitro­methyl­idene)imidazolidin-1-yl]ethanol

**DOI:** 10.1107/S1600536810039280

**Published:** 2010-10-09

**Authors:** Gaolei Wang, Dongmei Li, He Li

**Affiliations:** a Shandong Provincial Key Laboratory of Fluorine Chemistry and Chemical Materials, School of Chemistry and Chemical Engineering, University of Jinan, People’s Republic of China

## Abstract

In the title compound, C_6_H_11_N_3_O_3_, the imidazolidine NH group is involved in a three-center N—H⋯O hydrogen bond, with intra­molecular and inter­molecular branches, to the nitro group O atoms. The centrosymmetric dimers that are formed are further connected by O—H⋯O hydrogen bonds between the hy­droxy and nitro groups into a two-dimensional polymeric structure extending parallel to (101).

## Related literature

For related structures, see: Tian *et al.* (2010 ▶); Li *et al.* (2010 ▶). For background to neonicotinoid insecticides, see: Ohno *et al.* (2009 ▶); Jeschke & Nauen (2008 ▶).
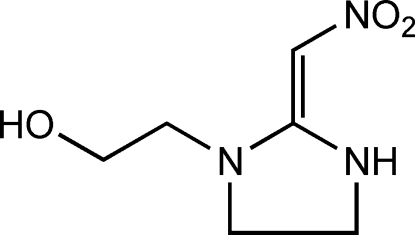


## Experimental

### 

#### Crystal data

C_6_H_11_N_3_O_3_
*M* *_r_* = 173.18
Monoclinic, 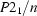

*a* = 6.9422 (2) Å
*b* = 8.7142 (3) Å
*c* = 12.9698 (4) Å
β = 94.153 (3)°
*V* = 782.55 (4) Å^3^
*Z* = 4
Mo *K*α radiation
μ = 0.12 mm^−1^
*T* = 293 K
0.31 × 0.29 × 0.25 mm

#### Data collection

Bruker APEXII CCD diffractometer
Absorption correction: multi-scan (*SADABS*; Bruker, 2005 ▶) *T*_min_ = 0.967, *T*_max_ = 1.0
4832 measured reflections
1539 independent reflections
1186 reflections with *I* > 2σ(*I*)
*R*_int_ = 0.017

#### Refinement

*R*[*F* ^2^ > 2σ(*F* ^2^)] = 0.038
*wR*(*F* ^2^) = 0.108
*S* = 1.11
1539 reflections
110 parameters
H-atom parameters constrained
Δρ_max_ = 0.17 e Å^−3^
Δρ_min_ = −0.17 e Å^−3^

### 

Data collection: *APEX2* (Bruker, 2005 ▶); cell refinement: *SAINT* (Bruker, 2005 ▶); data reduction: *SAINT*; program(s) used to solve structure: *SIR97* (Altomare *et al.*, 1999 ▶); program(s) used to refine structure: *SHELXL97* (Sheldrick, 2008 ▶); molecular graphics: *SHELXTL* (Sheldrick, 2008 ▶); software used to prepare material for publication: *WinGX* (Farrugia, 1999 ▶).

## Supplementary Material

Crystal structure: contains datablocks I, global. DOI: 10.1107/S1600536810039280/gk2305sup1.cif

Structure factors: contains datablocks I. DOI: 10.1107/S1600536810039280/gk2305Isup2.hkl

Additional supplementary materials:  crystallographic information; 3D view; checkCIF report

## Figures and Tables

**Table 1:** Hydrogen-bond geometry (Å, °)

*D*—H⋯*A*	*D*—H	H⋯*A*	*D*⋯*A*	*D*—H⋯*A*
N2—H2⋯O2^i^	0.86	2.37	3.0463 (16)	136
N2—H2⋯O2	0.86	2.12	2.6600 (16)	121
O1—H1⋯O3^ii^	0.82	2.06	2.8814 (16)	175

Symmetry codes: (i) 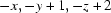; (ii) 
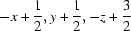
.

**Fig. 1.**
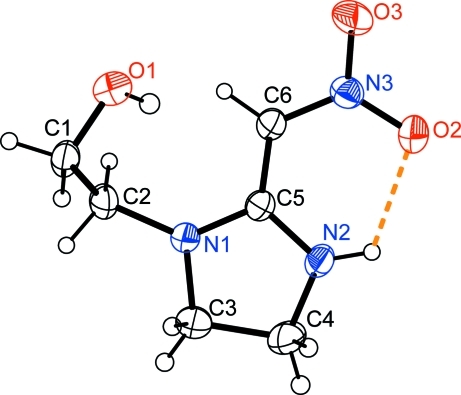
The molecular structure of the title compound with atom numbering scheme. Displacement ellipsoids are drawn at the 40% probability level. The H atoms are shown as spheres of arbitrary size.

**Fig. 2.**
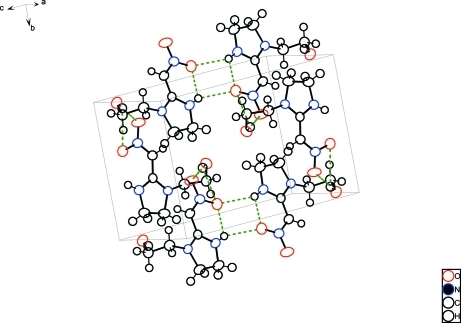
Inter- and intramolecular hydrogen bonding in the titlecrystal structure.

## supplementary crystallographic information

### Comment

Compared with conventional insecticides, nicotinoid insecticides have rapidly grown and become an important chemical class of insecticides in recent years because of their novel structure and mode of action (Ohno *et al.*, 2009 and Jeschke *et al.*, 2008). Here, we have synthesized a new compound by introducing an oxygen atom into the lead struture instead of nitrogen atom.

The structure of the title compound is shown in Fig. 1 with the atom-numbering scheme. The title compound is homolog of (*E*)-1-(2,2-dimethoxyethyl)-2-(nitromethylene)imidazolidine (Li *et al.*, 2010). The imidazolidine ring is close to planar (r.m.s. deviation = 0.006 Å). Intramolecular H-bonding of N–H···O type exists and completes an S(6) ring motif. The packing of the molecules is stabilized by N–H···O and O–H···O hydrogen bonds and van der Waal's forces.

### Experimental

A solution of 2-(2-aminoethylamino)ethanol (2 mmol), and 1,1-bis(thiomethyl)-2-nitroethylene (2 mmol) in 30 ml of ethanol was refluxed for 8 h and then cooled to room temperature. Evaporation under reduced pressure gave the title product after purifiction by flash chromatography. Single crystals suitable for X-ray analysis were obtained by slow evaporation of a solution of dichloromethane and ethyl acetate of the title compound.

### Refinement

All H atoms were placed in their calculated positions and then refined using riding model with C—H = 0.93–0.97 Å, O—H = 0.82 Å , N—H = 0.86 Å and *U*_iso_(H) = 1.2 *U*_eq_(C,N) or *U*_iso_(H) = 1.5 *U*_eq_(O).

### Crystal data

**Table:**

C_6_H_11_N_3_O_3_	*F*(000) = 368
*M**_r_* = 173.18	*D*_x_ = 1.478 Mg m^−^^3^
Monoclinic, *P*2_1_/*n*	Mo *K*α radiation, λ = 0.7107 Å
Hall symbol: -P 2yn	Cell parameters from 2585 reflections
*a* = 6.9422 (2) Å	θ = 3.2–28.8°
*b* = 8.7142 (3) Å	µ = 0.12 mm^−^^1^
*c* = 12.9698 (4) Å	*T* = 293 K
β = 94.153 (3)°	Prism, colourless
*V* = 782.55 (4) Å^3^	0.31 × 0.29 × 0.25 mm
*Z* = 4	

### Data collection

**Table:**

Bruker APEXII CCD diffractometer	1539 independent reflections
Radiation source: fine-focus sealed tube	1186 reflections with *I* > 2σ(*I*)
graphite	*R*_int_ = 0.017
Detector resolution: 16.0355 pixels mm^-1^	θ_max_ = 26.0°, θ_min_ = 3.2°
ω scans	*h* = −8→8
Absorption correction: multi-scan (*SADABS*; Bruker, 2005)	*k* = −10→10
*T*_min_ = 0.967, *T*_max_ = 1.0	*l* = −15→15
4832 measured reflections	

### Refinement

**Table:**

Refinement on *F*^2^	Primary atom site location: structure-invariant direct methods
Least-squares matrix: full	Secondary atom site location: difference Fourier map
*R*[*F*^2^ > 2σ(*F*^2^)] = 0.038	Hydrogen site location: inferred from neighbouring sites
*wR*(*F*^2^) = 0.108	H-atom parameters constrained
*S* = 1.11	*w* = 1/[σ^2^(*F*_o_^2^) + (0.0606*P*)^2^ + 0.045*P*] where *P* = (*F*_o_^2^ + 2*F*_c_^2^)/3
1539 reflections	(Δ/σ)_max_ < 0.001
110 parameters	Δρ_max_ = 0.17 e Å^−^^3^
0 restraints	Δρ_min_ = −0.17 e Å^−^^3^

### Special details

**Table:**

Geometry. All e.s.d.'s (except the e.s.d. in the dihedral angle between two l.s. planes) are estimated using the full covariance matrix. The cell e.s.d.'s are taken into account individually in the estimation of e.s.d.'s in distances, angles and torsion angles correlations between e.s.d.'s in cell parameters are only used when they are defined by crystal symmetry. An approximate (isotropic) treatment of cell e.s.d.'s is used for estimating e.s.d.'s involving l.s. planes.
Refinement. Refinement of *F*^2^ against ALL reflections. The weighted *R*-factor *wR* and goodness of fit *S* are based on *F*^2^, conventional *R*-factors *R* are based on *F*, with *F* set to zero for negative *F*^2^. The threshold expression of *F*^2^ > σ(*F*^2^) is used only for calculating *R*-factors(gt) *etc*. and is not relevant to the choice of reflections for refinement. *R*-factors based on *F*^2^ are statistically about twice as large as those based on *F*, and *R*- factors based on ALL data will be even larger.

### Fractional atomic coordinates and isotropic or equivalent isotropic displacement parameters (Å^2^)

**Table:**

	*x*	*y*	*z*	*U*_iso_*/*U*_eq_	
C1	0.7352 (2)	0.66673 (18)	0.73309 (13)	0.0428 (4)	
H1A	0.7010	0.7696	0.7099	0.051*	
H1B	0.8709	0.6509	0.7230	0.051*	
C2	0.70406 (19)	0.65267 (18)	0.84644 (12)	0.0387 (4)	
H2A	0.7338	0.5485	0.8687	0.046*	
H2B	0.7932	0.7207	0.8851	0.046*	
C3	0.4516 (2)	0.84632 (17)	0.89333 (14)	0.0481 (4)	
H3A	0.4536	0.9106	0.8324	0.058*	
H3B	0.5364	0.8904	0.9483	0.058*	
C4	0.2490 (3)	0.82924 (18)	0.92650 (15)	0.0519 (5)	
H4A	0.1570	0.8840	0.8802	0.062*	
H4B	0.2396	0.8668	0.9964	0.062*	
C5	0.36609 (19)	0.59107 (16)	0.88760 (10)	0.0296 (3)	
C6	0.3790 (2)	0.43186 (17)	0.87112 (11)	0.0358 (4)	
H6	0.4933	0.3920	0.8490	0.043*	
N1	0.50847 (16)	0.68944 (13)	0.87087 (9)	0.0336 (3)	
N2	0.21646 (17)	0.66554 (14)	0.92072 (10)	0.0403 (3)	
H2	0.1119	0.6216	0.9370	0.048*	
N3	0.23318 (18)	0.33545 (14)	0.88605 (10)	0.0371 (3)	
O1	0.62388 (16)	0.55935 (12)	0.67291 (8)	0.0481 (3)	
H1	0.5161	0.5949	0.6580	0.072*	
O2	0.07390 (15)	0.38210 (13)	0.91700 (9)	0.0480 (3)	
O3	0.25342 (18)	0.19348 (13)	0.86750 (10)	0.0580 (4)	

### Atomic displacement parameters (Å^2^)

**Table:**

	*U*^11^	*U*^22^	*U*^33^	*U*^12^	*U*^13^	*U*^23^
C1	0.0326 (8)	0.0405 (9)	0.0571 (10)	−0.0014 (6)	0.0154 (7)	0.0017 (7)
C2	0.0258 (7)	0.0403 (9)	0.0499 (9)	−0.0013 (6)	0.0021 (6)	−0.0009 (7)
C3	0.0482 (10)	0.0322 (9)	0.0651 (11)	−0.0019 (7)	0.0120 (8)	−0.0064 (7)
C4	0.0552 (11)	0.0338 (9)	0.0689 (12)	0.0060 (7)	0.0210 (9)	−0.0038 (7)
C5	0.0292 (7)	0.0324 (8)	0.0274 (7)	0.0016 (6)	0.0026 (5)	0.0012 (5)
C6	0.0303 (8)	0.0324 (8)	0.0454 (8)	0.0010 (6)	0.0077 (6)	−0.0015 (6)
N1	0.0319 (6)	0.0298 (7)	0.0399 (7)	−0.0019 (5)	0.0087 (5)	−0.0032 (5)
N2	0.0329 (7)	0.0329 (7)	0.0568 (8)	0.0031 (5)	0.0162 (6)	−0.0007 (5)
N3	0.0389 (7)	0.0323 (7)	0.0400 (7)	−0.0019 (5)	0.0023 (5)	−0.0002 (5)
O1	0.0507 (7)	0.0442 (7)	0.0502 (7)	0.0038 (5)	0.0083 (5)	−0.0071 (5)
O2	0.0372 (6)	0.0480 (7)	0.0606 (7)	−0.0060 (5)	0.0163 (5)	−0.0029 (5)
O3	0.0593 (8)	0.0293 (7)	0.0858 (9)	−0.0038 (5)	0.0090 (7)	−0.0056 (6)

### Geometric parameters (Å, °)

**Table:**

C1—H1A	0.9700	C5—C6	1.408 (2)
C1—H1B	0.9700	C6—H6	0.9300
C1—C2	1.506 (2)	N1—C2	1.4523 (17)
C2—H2A	0.9700	N1—C3	1.4582 (19)
C2—H2B	0.9700	N2—H2	0.8600
C3—H3A	0.9700	N2—C4	1.445 (2)
C3—H3B	0.9700	N3—O3	1.2701 (16)
C3—C4	1.508 (2)	N3—C6	1.3406 (18)
C4—H4B	0.9700	O1—H1	0.8200
C4—H4A	0.9700	O1—C1	1.4123 (19)
C5—N1	1.3379 (17)	O2—N3	1.2702 (15)
C5—N2	1.3227 (17)		
			
C1—O1—H1	109.5	N1—C5—C6	123.43 (13)
C1—C2—H2A	108.9	N1—C2—C1	113.42 (12)
C1—C2—H2B	108.9	N1—C2—H2A	108.9
H1A—C1—H1B	107.9	N1—C2—H2B	108.9
C2—N1—C3	121.44 (12)	N1—C3—H3A	111.0
C2—C1—H1A	109.2	N1—C3—H3B	111.0
C2—C1—H1B	109.2	N1—C3—C4	103.67 (12)
H2A—C2—H2B	107.7	N2—C5—N1	110.19 (12)
C3—C4—H4B	111.1	N2—C5—C6	126.39 (13)
C3—C4—H4A	111.1	N2—C4—C3	103.18 (12)
H3A—C3—H3B	109.0	N2—C4—H4B	111.1
C4—N2—H2	123.9	N2—C4—H4A	111.1
C4—C3—H3A	111.0	N3—C6—C5	122.59 (13)
C4—C3—H3B	111.0	N3—C6—H6	118.7
H4B—C4—H4A	109.1	O1—C1—H1A	109.2
C5—N1—C2	127.40 (12)	O1—C1—H1B	109.2
C5—N1—C3	110.75 (12)	O1—C1—C2	111.96 (12)
C5—N2—H2	123.9	O2—N3—C6	121.94 (12)
C5—N2—C4	112.19 (12)	O3—N3—O2	118.84 (12)
C5—C6—H6	118.7	O3—N3—C6	119.21 (13)
			
O1—C1—C2—N1	−64.63 (17)	C2—N1—C3—C4	−173.37 (14)
O2—N3—C6—C5	−0.6 (2)	C3—N1—C2—C1	−87.91 (16)
O3—N3—C6—C5	178.47 (14)	C5—N1—C2—C1	100.19 (16)
N1—C5—N2—C4	1.41 (17)	C5—N1—C3—C4	−0.24 (17)
N1—C5—C6—N3	−178.43 (12)	C5—N2—C4—C3	−1.48 (19)
N1—C3—C4—N2	0.97 (18)	C6—C5—N1—C2	−8.2 (2)
N2—C5—N1—C2	171.93 (13)	C6—C5—N1—C3	179.16 (14)
N2—C5—N1—C3	−0.69 (16)	C6—C5—N2—C4	−178.44 (14)
N2—C5—C6—N3	1.4 (2)		

### Hydrogen-bond geometry (Å, °)

**Table:**

*D*—H···*A*	*D*—H	H···*A*	*D*···*A*	*D*—H···*A*
N2—H2···O2^i^	0.86	2.37	3.0463 (16)	136
N2—H2···O2	0.86	2.12	2.6600 (16)	121
O1—H1···O3^ii^	0.82	2.06	2.8814 (16)	175

Symmetry codes: (i) −*x*, −*y*+1, −*z*+2; (ii) −*x*+1/2, *y*+1/2, −*z*+3/2.
